# Characterization of a Diverse Okra (*Abelmoschus esculentus* L. Moench) Germplasm Collection Based on Fruit Quality Traits

**DOI:** 10.3390/plants14040565

**Published:** 2025-02-12

**Authors:** Mehtap Yildiz, Sibel Turan Sirke, Metin Koçak, İbrahim Mancak, Aslıhan Agar Özkaya, Kazım Abak, Okan Özkaya, Pablo Federico Cavagnaro

**Affiliations:** 1 Department of Agricultural Biotechnology, Faculty of Agriculture, Van Yuzuncu Yil University, Van 65080, Turkey; sibelturan@yyu.edu.tr (S.T.S.); metinkocak@yyu.edu.tr (M.K.); 2 Manier Seed Company, Adana 01355, Turkey; ibrahim@manier.com.tr; 3 Department of Horticulture, Faculty of Agriculture, Cukurova University, Adana 01330, Turkey; aozkaya@cu.edu.tr (A.A.Ö.); kazimabak@gmail.com (K.A.); okanozkaya@yahoo.com (O.Ö.); 4 Consejo Nacional de Investigaciones Científicas y Técnicas (CONICET), Ciudad Autonoma de Buenos Aires C1425FQB, Argentina; 5 Instituto Nacional de Tecnología Agropecuaria (INTA), Estación Experimental Agropecuaria Mendoza, Luján de Cuyo M5534, Argentina; 6 Department of Plant Biology and Biotechnology, Faculty of Biotechnology and Horticulture, University of Agriculture in Krakow, 31-120 Krakow, Poland

**Keywords:** *Abelmoschus esculentus*, germplasm characterization, fruit quality, vitamin C, chlorophyll content

## Abstract

Okra is an important dietary component of many Asian countries, providing high levels of dietary fiber, phytonutrients (e.g., antioxidant vitamins and pigments), and essential minerals. Evaluation of okra germplasm collections can improve the curation of genebanks and help identify superior materials for breeding. In this study, 66 okra accessions from diverse geographical origins were characterized based on fruit quality traits, including fruit fresh (FFW) and dry weights (FDW), dry matter (DM), diameter, length, and diameter of the fruit peduncle; concentration of vitamin C, chlorophyll a and b, and total chlorophyll; and color-chroma values. Significant (*p* < 0.05) and substantial variation was found among the accessions for all traits. Mean FFW and FDW varied nearly three-fold, with ranges of 3.76–9.99 g and 0.43–1.34 g, respectively, with a range in DM content of 10.5–19.4%. Vitamin C and total chlorophyll content varied 6.4- and 8.3-fold, with ranges of 12.8–82.8 and 1.07–8.91 mg/100 g fw, respectively, with substantial variation also observed in chlorophyll composition. Significant positive correlations were found between vitamin C and total and subtypes of chlorophyll levels (r = 0.29–0.32), whereas the strongest correlations were between FFW and FDW (r = 0.88) and between total chlorophyll and chlorophyll subtypes a and b (r = 0.90–0.95). Additionally, a dendrogram constructed based on these phenotypic data grouped the accessions in general agreement with their geographical origins and fruit traits. Overall, our results revealed broad phenotypic diversity in the evaluated germplasm, which is exploitable in okra breeding programs aimed at increasing fruit quality and nutraceutical value.

## 1. Introduction

Okra (*Abelmoschus esculentus* L. Moench) is an important vegetable belonging to the Malvaceae family. It originated in Africa, but it is also cultivated under subtropical, tropical, and warm climatic conditions. Okra can be grown in a wide range of soils under rain-fed or irrigated conditions. Due to its heat tolerance and rusticity, okra does not require high-level technology for its cultivation [1]. In Asian countries, where it is mostly consumed, it plays an important role in human nutrition, as it contains essential dietary components such as healthy carbohydrates (e.g., soluble and insoluble fiber), minerals, antioxidant vitamins, proteins, folic acid, phosphorus, magnesium, calcium, and potassium [2].

In Turkey, which is one of the world’s okra producers and consumers, okra is grown in all regions except the Eastern and Northeastern provinces [1]. These productions generally use local populations and varieties, which are phenotypically distinct and tend to have—within each variety—a homogeneous appearance of the fruits due to the small local harvest and seed production, with fruits having quality and flavor characteristics that are suitable for the Turkish market and local consumer preferences. Conversely, other okra traits exhibit a wide range of variation across varieties and populations [3,4]. In one of these studies, red pigmentation in various plant parts, degree of lateral branching, and flowering behavior served as distinguishing phenotypic traits for accessions of diverse origins, whereas, for Turkish accessions, no common distinctive trait was evident [5]. Analyses of genetic relationships and clustering among okra accessions from different ecogeographical regions and their transitions are scarce. Vural et al. [6] suggested that four cultivars were responsible for most of the genetic diversity in the Turkish germplasm, namely the Sultani group, Balikesir, Bornova, and Amasya. Another study examined 21 morphological traits, which were used to perform clustering analysis in a collection of Turkish okra landraces, revealing 10 subtypes of okra according to their adaptation to the territorial Anatolian, Western-Trace, and Aegean climates [1]. Olivera et al. [7] classified okra fruits belonging to different local varieties and introduced cultivars (Emerald, Clemson Spineless, and Annie Oakley II), reporting that local varieties were firm although smaller, whereas the hybrid material (Annie Oakley II) yielded high-quality fruits with greater dry matter content (DM). They also reported that the concentration of total phenolics significantly increased with fruit size in local varieties, whereas no significant differences were found among Annie Oakley II samples.

From a breeding perspective, it is important to develop okra segregating populations with a broad genetic basis from which the desired traits can be selected for use in breeding programs. A prerequisite to this end is the characterization of the available genetic resources for traits of interest and, after that, attempting directed crosses in order to transfer such traits in the desired genetic background [8]. In recent years, the production and consumption of most vegetable crops have increased, and okra breeding programs have accelerated, aiming to satisfy consumer needs and preferences. Morphological, genetic, and cytogenetic analyses of the germplasm resources within a species, followed by their classification and evaluation of the genetic relationships among them, will likely improve the success rate of a breeding program [9]. The quality traits of okra pods are significantly influenced by the cultivar. Various studies have documented the nutritional, biochemical, and physical characteristics of okra pods, highlighting the importance of cultivar selection for improving these attributes in breeding programs. For instance, the nutritional assessment of different okra cultivars revealed that mean protein, ash, and fiber contents varied significantly among the studied plant materials, with certain high-yield cultivars, like IHR-31, exhibiting superior nutrient profiles compared to others, suggesting that cultivar choice can strongly influence the nutritional value of the pods [10]. Okra is also a good source of vitamins A, B, and C. Fresh pods are low in calories (~20 g per 100 g of fresh weight), contain negligible amounts of fat (0.2 g per 100 g fw), and are high in fiber and other valuable nutrients, including ~30% of the recommended daily intake of vitamin C (16–29 mg), 10–20% of folate (46–88 μg), and about 5% of vitamin A (14-20 RAE (retinol activity equivalent)) [11]. In addition, the consumption of dark green vegetables with high chlorophyll content has been shown to be beneficial for human health, as chlorophyll possesses antioxidant and anti-carcinogenic properties. From a breeding perspective, increasing pod nutritional value would directly benefit okra consumers and may provide access—for okra growers—to niche markets with increased health awareness.

Plant attributes such as leaf morphology, root structure, flower color, and fruit shape represent the basic traits and criteria used by the International Union for the Protection of New Varieties of Plants (UPOV) for the classification of okra germplasm resources. Analysis of morphological traits—if performed at the same plant developmental stage and under the same environmental conditions and agricultural practices—is a relatively straightforward, reliable, and cost-effective method for germplasm characterization [12]. In addition, morphological characterizations are important for the development of productive and high-quality varieties that can adapt to specific environments, such as those affected by a range of abiotic stress (e.g., drought, salinity, high temperatures, etc.) [13]. Although Turkey is not the center of origin of okra, its germplasm harbors ample genetic and phenotypic variation [5,14], presumably due to successive plant introductions, local selections, low-scale breeding, and cultivation that took place in the country throughout history.

The improvement of fruit quality traits, such as fruit size and shape and dry matter content, plays a crucial role in okra productivity and marketability [15]. Defined criteria for okra’s fruit morphological parameters are not completely clear, but the United States Department of Agriculture (USDA) indicated pod length as a relevant trait to discriminate among okra accessions [7]. Other valuable traits that characterize okra fruits are diameter, greenness, mucilage, and fiber content [7]. According to Martin et al. [16], fruit greenness, length, and weight are all genotype-dependent traits. Although industrial preference demands long or medium-sized fruits, smaller fruits are frequently preferred by consumers. Greenness and fruit diameter are also important quality traits, and being very dark green and reduced fruit diameter are generally preferred [7,15].

Although previous studies have characterized okra germplasm, they used few accessions and/or phenotypic traits, mostly focusing on different plant traits [1,4,5,7,8,9,10]. Thus, evaluation of a broad germplasm collection from diverse geographical origins using a relatively large number of agriculturally and nutritionally relevant fruit traits may provide a more robust assessment of the genetic diversity—for traits of the consumed organ—within this species. In addition, it may help to identify redundancies and verify uniqueness within the evaluated germplasm, thereby increasing the efficiency of collection conservation efforts. In this study, we characterized a large and phenotypically diverse okra germplasm collection (66 accessions) derived from various geographical origins for 13 fruit morphometric and quality traits, including fresh and dry weights, dry matter content, fruit diameter and length, peduncle diameter, concentration of vitamin C, total chlorophyll, chlorophylls a and b, and three color measurements (hue angle, luminance, and chroma index). The phenotypic data were used to (1) identify superior accessions with high quality and nutritional value; (2) investigate possible associations among the variables by means of correlation analysis; and (3) reveal genetic relationships among the accessions based on clustering analysis. The resulting data will be of value for okra’s breeding programs.

## 2. Results and Discussion

### 2.1. Phenotypic Variation Based on Morphological, Biochemical, and Color Traits of Okra Fruits

Determination of quality parameters in the available okra germplasm is a prerequisite for identifying and selecting superior accessions to be used in breeding programs. In this study, we analyzed fruit fresh weight (FFW), dry weight (FDW), DM, diameter (FD), length (FL), and peduncle diameter (PD) (among other traits), which are important breeding traits, as they represent major fruit quality attributes that are valued by both the okra market and consumers. In addition, FFW is one of the most important yield components, and this is particularly relevant in okra, which forms fruits in each leaf axil [17]. As shown in Table 1, significant variation (*p* ≤ 0.05) was found among the accessions for all of these traits.

FFW varied nearly three-fold in the okra germplasm, with values ranging from 3.8 (in accession IN-7) to 10.0 g (in AF-3) and an overall mean of 5.4 g for the entire collection. The reported variation is mainly due to genetic differences among the accessions since the fruits were harvested and weighted at the same plant developmental stage and fruit maturity for all accessions, which were grown under the same environmental conditions and agricultural practices. In comparison to our results, Nwangburuka et al. [18] obtained similar figures for this trait, reporting a range of FFW of 5.6–11.7 g, with an overall mean of 7.8 g, among 29 Nigerian okra accessions. A narrower range of variation with comparable mean FFW value was found in a study by Düzyaman [1], which analyzed 10 Turkish okra cultivars, reporting a range of 3.9–6.3 g and an overall mean of 5.3 g. Similarly, Ece et al. [4] reported a smaller FFW range of 3.6–5.7 g for okra germplasm from the Turkish region of Amasya. In contrast, a study using Indian okra germplasm reported a greater overall mean and range for FFW, with values of 14.4 g and 10.8–20.6 g, respectively [19]. Differences in the extent of genetic variation found for FFW among these studies may be attributable to differences in the type and number of the accessions analyzed, the environmental conditions and agricultural practices under which the plants were grown (e.g., fertilization and watering regimes), and/or the degree of fruit maturity and harvest time used to collect fruit samples and measure FFW.

In the present study, FDW varied approximately three-fold among the accessions, with a range and overall mean of 0.43–1.34 g and 0.67 g, respectively. TR-33-1 and AF-5 were the accessions with the lowest and highest mean FDW values, respectively (Table 1). DM, expressed as a percentage of total fresh weight, is an important trait for the dehydration industry, as the greater the DM, the less amount of energy is required for dehydration, and it is positively associated with the postharvest shelf-life of many horticultural fruit products, including okra [1]. In the present work, DM varied nearly two-fold, from 10.5% in accession A-8 to 19.6% in TR-35-1. In comparison, Düzyaman (2005) [1] found a much smaller level of variation for this trait among 10 Turkish okra cultivars, reporting a range of DM of 12.1–13.6% and a mean of 13.0%. The broader range of variation found for this trait in our study is likely due to the larger and genetically more diverse germplasm used herein (i.e., 66 accessions from eight countries and four continents).

FL, an important parameter for okra breeding and commercial markets, varied more than two-fold, exhibiting a range of mean values from 28.5 mm in accession TR-54-1 to 63.1 mm in TR-60-1, with an overall mean of ~45.0 mm (Table 1). The ranges of variation found for this trait in previous studies and in the present work differ greatly. For example, Demikir [17] reported lower FL values in okra materials from Amasya (Turkey), with a range of 11–31 mm. Conversely, Binalfew and Alemu [20] found a much greater degree of FL variation in okra germplasm from western Ethiopia, with a reported range of 51–193 mm. Similarly, Younis et al. [21] and Nwangburuka et al. [18] also indicated broader FL ranges with higher upper limits, namely 32.0–138.1 and 74.5–204.2 mm for Egyptian and Nigerian okra germplasms, respectively. Lastly, comparable values to our data were reported by Eshiet and Brisibe [8], who indicated an FL range of 32.3–68.3 mm in selected okra varieties. The discrepancies among these studies may be due to differences in the type and amount of genetic stocks analyzed and/or the environmental conditions and agricultural practices used among the studies.

FD, another relevant trait associated with fruit quality, varied nearly two-fold in our okra germplasm and ranged from 9.3 mm in accession Akkoy to 19.0 mm in AF-3, with an overall mean of 11.8 mm (Table 1). These figures are lower than those found in a study that evaluated 50 okra accessions from Ethiopia, reporting an FD range of 16–43 mm and an overall mean of 27.3 mm [20]. It is likely that such discrepancies may be due to differences in the germplasm evaluated in both studies, perhaps as a result of a stronger preference for thicker pods among Ethiopian growers, as the accessions used in their study were selected from major okra production regions of Ethiopia. PD varied nearly two-fold, between 4.0 and 7.3 mm, with a mean of 5.3 mm. TR-47-1 and ‘Cyprus’ were the accessions with the lowest and greatest mean values. To the best of our knowledge, no other reports have evaluated this trait in okra germplasm.

Results for total chlorophyll content (TCC), chlorophyll composition (as estimated by determination of chlorophyll fractions a and b), and vitamin C levels, along with fruit color parameters, are presented in Table 2. Significant (*p* ≤ 0.05) and substantial variation was found among the accessions for all these traits, as estimated by coefficient of variation (CV) values in the range of 34.8–65.6% and 12.2–60.9% found for biochemical and color parameters, respectively (Table 2). In okra, TCC reflects the fruit nutritional status and overall plant health. TCC in fresh okra fruits, assessed immediately after harvest, varied ~8.3-fold, with a range of 1.07-8.91 mg/100 g fw and an overall mean of 3.62 mg/100 g fw. The accessions Cyprus and TR-42-1 had the lowest and greatest TCC, respectively. In nearly all the accessions (with the single exception being TR-05-1), chlorophyll a predominated over chlorophyll b, with the former fraction contributing, on average for all the accessions, to 70.4% of the TCC (range was 42.5–95.5%). In absolute values, chlorophyll a content (CAC) varied 6.9-fold among the accessions and ranged from 0.76 mg/100 g fw in TR-10-2 to 5.25 mg/100 g fw in TR-42-1, with an overall mean of 2.5 mg/100 g fw. Chlorophyll b content (CBC) represented a smaller chlorophyll fraction, accounting—on average—for 29.4% of the TCC (range was 4.5–57.5%). In absolute values, CBC varied 17.6-fold among the accessions and ranged from 0.23 mg/100 g fw in IN-8 to 4.06 mg/100 g fw in TR-42-1, with a general mean of 1.14 mg/100 g fw. Thus, altogether, our results indicate TR-42-1 as the accession with the greatest TCC, CAC, and CBC, whereas three accessions—namely Cyprus, TR-10-2, and IN-8—conformed to the low end of these variables, respectively. The broad variation found in this germplasm collection for TCC as well as for chlorophyll composition, as estimated by CAC and CBC absolute and relative values, indicate the availability of valuable genetic resources that can be used in breeding programs for increasing okra’s functional properties, highlighting TR-42-1 as a superior genotype for this purpose. In addition, these contrasting materials could be used to develop segregating populations for QTL mapping and analysis of candidate genes, conditioning chlorophyll content and composition.

Chlorophyll plays a significant role in the plant’s ability to convert light energy into chemical energy, thus influencing growth and yield. As shown in this study, TCC can vary significantly and broadly among okra accessions (Table 2). In comparison, a previous study analyzed a single Tunisian okra variety, finding mean TCC, CAC, and CBC levels of 6.02, 3.53, and 2.43 mg/100 g fw, respectively [22], all of which fall within the range of values found in the present work for these variables. Another study analyzed eight Mediterranean okra cultivars and landraces, reporting ranges of values for TCC, CAC, and CBC of 0.68–2.26, 0.38–1.43, and 0.22–0.83 mg/100 g fw, respectively [23]. The generally lower values and narrower ranges reported in the latter work, as compared to our data (i.e., 1.07–8.91, 0.76–5.25, and 0.23–4.06 mg/100 g fw, for TCC, CAC, and CBC, respectively; Table 2) are likely due to the larger and more diverse germplasm collection used in the present study. In addition to genotype, chlorophyll content and composition can also be affected by environmental factors such as soil fertility, water availability, and phytopathogen incidence. For example, previous research indicates that chlorophyll content in okra can be increased by fertilization, using different types and treatments of chemical and organic fertilizers [2,24]. This suggests that cultivar selection and fertilization strategies can be optimized to increase chlorophyll levels, thereby potentially increasing photosynthetic efficiency and crop yield. Chlorophyll content positively influences the aesthetic quality of the pods and also serves as an indicator of plant health and senescence, which is crucial for consumer perception and market value.

Ascorbic acid, also called vitamin C, is an essential phytochemical of the plant antioxidant system, and its concentration in okra pods represents an important trait of nutritional value for consumers. In addition, vitamin C is involved in the biosynthesis of ethylene and in hormonal signaling in different plant growth and developmental processes, often in response to environmental cues. In the present study, vitamin C content in fresh fruits, as determined immediately after harvest, varied 6.4-fold, exhibiting mean values between 12.8 and 82.8 mg/100 g dw, with an overall mean of 46.8 mg/100 g dw. The greatest vitamin C content was found in accession TR-77-1, whereas TR-27-1 had the lowest content. In comparison, vitamin C levels in six commercial varieties from Côte d’Ivoire in western Africa ranged from 25.3 to 49.6 mg/100 g dw, with a mean of 39.6 mg/100 g dw [25]. Another study that compared okra germplasm from four regions of Egypt reported a range for vitamin C concentration of 11.6–27.1 mg/100 g dw, with a mean of 16.7 mg/100 g dw [26]. The broader range and greater overall mean found for vitamin C content in the present study, as compared to those reported by Combo et al. [25] and Sami et al. [26], are likely due to the much larger and diverse germplasm collections evaluated herein as compared to these previous reports (i.e., 66 vs. 6 vs. 4 accessions, respectively). The broad variation found for vitamin C levels in our germplasm collection will be useful in breeding programs aiming at increasing okra’s nutraceutical value, as well as for pursuing research to identify QTL and candidate genes conditioning this trait (e.g., by developing segregating populations followed by candidate gene analysis in the QTL region). The development of new cultivars with high vitamin C levels may be particularly relevant for some okra food products, such as sun-dried pods (mostly consumed during the off-season when fresh pods are not available), as the sun-drying process has been shown to significantly reduce vitamin C levels (i.e., ~46% of vitamin C content was lost in okra pods after the drying process) [27], and therefore using cultivars with high levels of this phytonutrient may help counteract part of its losses during the drying process.

**Figure 1:**
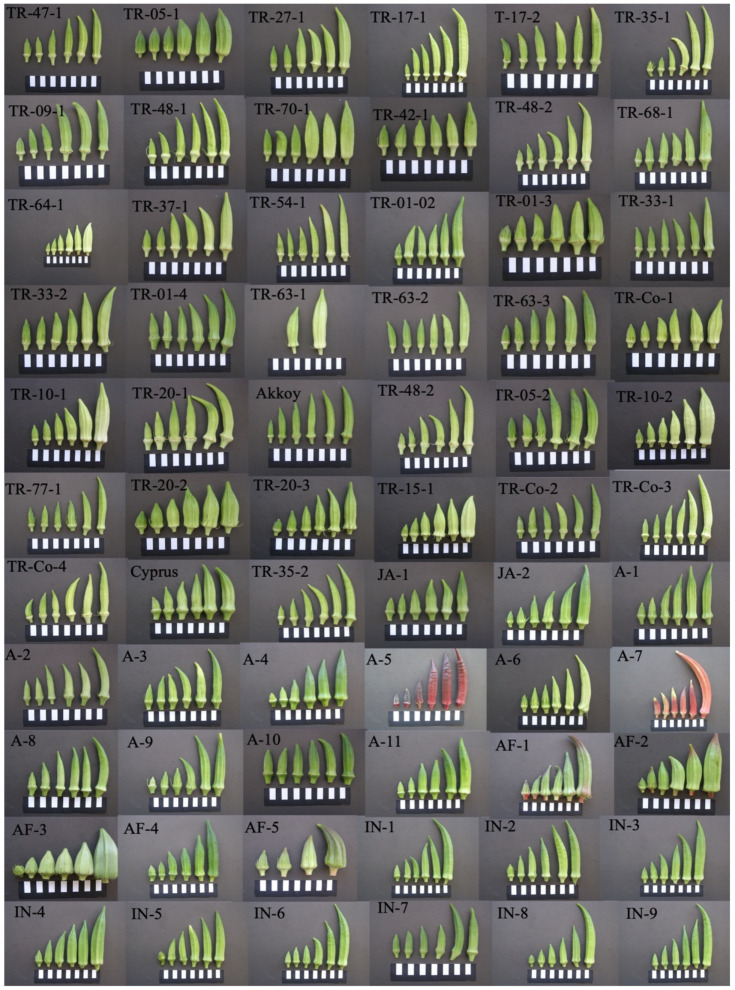
Fruit phenotypes at different developmental stages for 66 okra accessions. Developmental stages reflect different fruit lengths, in the range of 1.5–10.0 cm, with the last image to the right of the mature pod considered for phenotypic and biochemical characterization. The image with white rectangles was used as a size standard to compare pod sizes among images.

The intensity of green color in fruits is a quality trait for the okra market. Findings related to fruit color are presented in Table 2. The three color variables evaluated—namely luminance (L*), hue angle (Hue), and chroma index (Chr)—revealed significant variation among the okra accessions. Chroma values, expressing the saturation of color, varied ~3.6-fold, with a range of 239.5–862.1 and an overall mean of 629.7. The greatest chroma value was observed in TR-70-1, while the lowest value was in TR-15-1. Data for hue angle, which determines the quality of the color, varied 6.3-fold, with a range of 82.0–519.1 and a general mean of 113.6. In the majority of the evaluated germplasm (i.e., in 58 accessions, representing ~88% of the plant materials evaluated), fruit hue angle values were below 120, indicating light green to green color (Table 2, Figure 1). In contrast, the fruits of accessions A5 and A-7, which presented the greatest hue angle values (i.e., 452.08 and 519.11, respectively), were noticeably red due to the accumulation of anthocyanins in the fruit pericarp (Table 2 and Figure 1).

### 2.2. Relationships Among Okra Phenotypic Traits

Table 3 presents correlation coefficient (r) values among all the variables. The strongest positive correlations were found between FFW and FDW (r = 0.89, *p* < 0.001) and among total chlorophyll and chlorophyll subtypes a and b (r = 0.76–0.96, *p* < 0.001). Also, moderate positive relationships were found between fruit diameter, FFW, and FWD (r = 0.51–0.63, *p* < 0.001), whereas FDW was positively correlated with dry matter content (r = 0.38, *p* < 0.01). Together, these data suggest that the selection of accessions producing wider fruits with thicker pericarp may increase fruit weight and solids content. This may also be effective for increasing seed production yields, as strong positive correlations were previously reported between FFW and seed yield in okra accession from Ghana [28]. However, such an increase in individual fruit quality parameters may be at the expense of reducing total fruit yield per plant, as suggested by the moderate-to-strong negative associations (r = −0.56 to −0.83, *p* < 0.01) found previously between fruit weight and total fruit yield in okra parental lines, F_1_, and F_2_ populations [28]. In the present study, significant positive correlations were also found between peduncule diameter and FFW and FDW (r = 0.32–0.42, *p* < 0.05), as well as between peduncule diameter and fruit diameter (r = 0.27, *p* < 0.05). These associations make sense, as thicker peduncles are required to hold larger and heavier fruits. Our data also showed that fruit length was negatively—although weakly—correlated with fruit diameter (r = −0.25, *p* < 0.05), in agreement with a previous work reporting moderate-to-strong negative associations (r = −0.34 to −0.81, *p* < 0.01) between these two traits [29], suggesting an antagonistic relationship between the growth in length versus width of okra fruits.

Relationships among biochemical and color parameters were also examined. Interestingly, vitamin C content was positively correlated with TCC, CAC, and CBC (r = 0.29–0.32, *p* < 0.01), probably reflecting a general relationship between the health and nutritional status of the fruit (as indicated by chlorophyll concentration) and vitamin C levels. Among the color parameters, we found that luminance (L*) was negatively correlated with hue (r = −0.62, *p* < 0.01) and chromas (r = −0.40, *p* < 0.01), likely reflecting antagonistic relationships between green and red color intensities in okra fruits. Noteworthy, we found no significant correlations between biochemical and color parameters for most of the pair-wise trait comparisons, with only one exception, namely L* and CAC, for which a significant—yet weak—and a negative correlation was found (r = −0.26, *p* < 0.01).

### 2.3. Phenotypic and Genetic Relatedness Among the Okra Accessions

**Figure 2:**
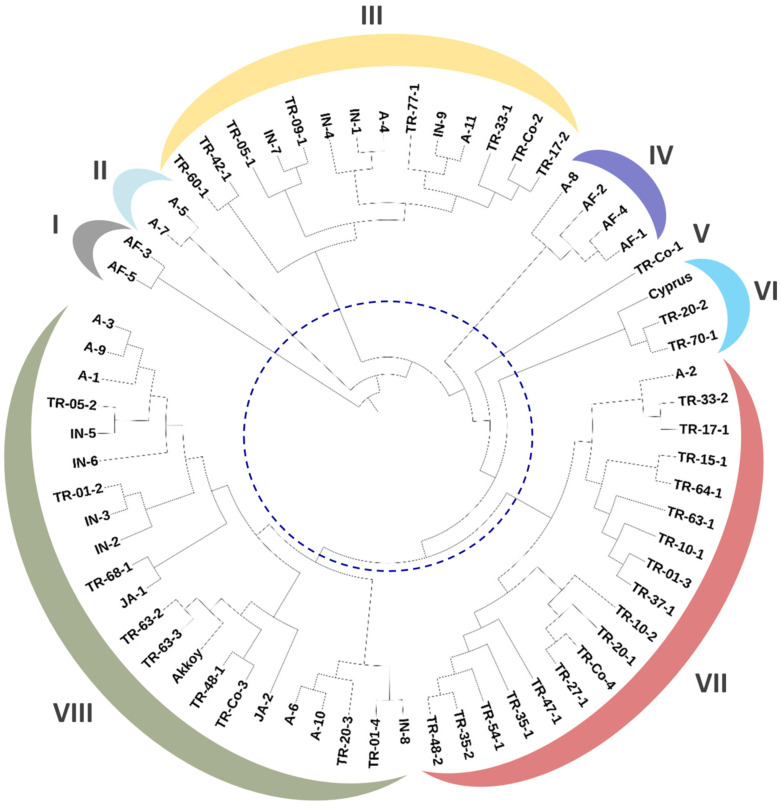
Unweighted pair group method with arithmetic means (UPGMA) dendrogram depicting phenotypic relationships among 66 okra accessions based on 13 fruit quality traits. Gower coefficient of similarity was used to establish pair-wise similarities among the taxa. The dashed blue line indicates delimitation of the eight phenotypic clusters denoted by colored arcs.

**Figure 3:**
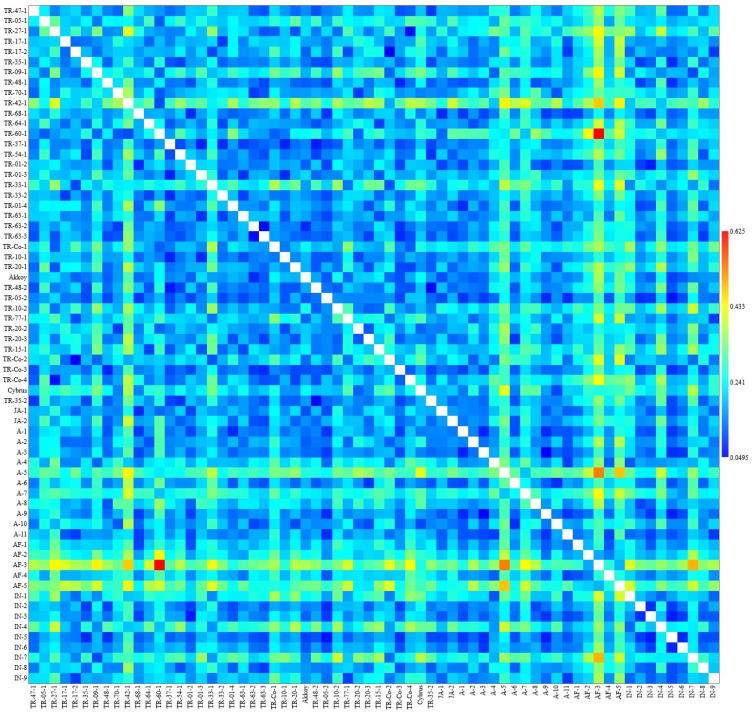
Heat map depicting pair-wise dissimilarity values among 66 okra accessions. The two-dimensional matrix reflects color-coded dissimilarity values (based on the Grower coefficient) among pair-wise accessions, with warmer colors indicating more phenotypically dissimilar pairs of accessions and colder colors indicating more similar accessions based on the fruit parameters evaluated in this study.

A dendrogram based on 13 fruit phenotypic traits for 66 okra accessions was generated according to the Gower coefficient of similarity (Figure 2). Eight phenotypic clusters were identified, designated as clusters I to VIII, with clustering of the accessions showing general agreement with their geographic origins (i.e., countries). Among the Turkish germplasm, 17 accessions were phenotypically closely related and were clustered together in clade VII, containing almost exclusively Turkish materials (the only exception was accession A2, from the USA), whereas the other Turkish accessions were included in clades III, V, VI, and VIII, together with plant materials from other geographic origins. Cluster I conformed to the outermost branch of the dendrogram and contained two accessions from Togo, namely AF-3 and AF-5, characterized by their thick and short pods of pale green color (Figure 1). Cluster II was also an external branch of the dendrogram and included only two accessions, A-5 and A7, the only two materials with red pods (Figure 1), also characterized for having the greatest hue values and the lowest luminance values in the entire germplasm collection. Clusters III and VIII contained mixed materials, mostly from Turkey, India, and the USA, indicating similar fruit phenotypes for these taxa regardless of their origin. The fact that Turkish materials were included in various clusters (clusters III, V–VIII) suggests broad phenotypic variation within the Turkish okra germplasm in terms of fruit quality parameters. These results are in agreement with the genetic clusters of Turkish okra germplasm obtained earlier using molecular markers [14]. A heat map depicting pair-wise phenotypic dissimilarities values (Grower coefficient) among the accessions evidenced TR-60-1 and AF-3 as the most distantly related accessions in terms of fruit phenotypes (Figure 3). The pair-wise dissimilarity coefficient for all the accessions ranged between 0.0495 and 0.0625.

Our previous studies evaluated genetic diversity in Turkish okra germplasm by means of sequence-related amplified polymorphism (SRAP) and peroxidase gene molecular marker analyses [5,14]. In these studies, clustering analyses separated—as plant materials that are genetically distinct from the rest—some of the same accessions that were found to be phenotypically different in the present work. One of these accessions was TR-10-2, characterized by bearing thick fruits with a pale green color and presenting one of the lowest chlorophyll contents (Table 2, Figure 1). Also, the two red-fruited accessions, A5 (in our previous study denominated as “Red Wonder”) and A7 (“UGA Red”), which in this work were found to have the greatest hue values and the lowest luminance (L*) values, were previously included in a separate and distinct genetic cluster conforming to one of the most external branches of the dendrogram in our molecular-based analysis [14]. In the present work, these two red-fruited accessions conformed to a clearly distinct—and one of the most external—phenotypic cluster of the dendrogram (Figure 2), evidencing the genetic and phenotypic uniqueness of these materials. Similarly, the two accessions from Togo, AF-3 (denominated as “1051 Togo” in our previous work) and AF-5 (“1159 Togo), characterized by their very thick and short pods of pale green color (Figure 1), were closely related both genetically and phenotypically, conforming to one of the most external clusters in both studies [14], and Figure 2 of this study.

## 3. Material and Methods

### 3.1. Plant Material

The plant material of this study consisted of 66 okra accessions, of which 38 were of Turkish origin, and the remaining 28 were from diverse countries of Asia, Africa, Europe, and America (Table 4 and Figure 1). The Turkish accessions can be regarded as current local landraces. Okra seeds were sawn in pots in a greenhouse (seeds were not pre-treated by any means). When the plants had 3–4 true leaves, around mid-April, ten plants per accession were transplanted in an open field of the Cukurova University (Adana, Turkey), with a plant spacing of 20 × 75 cm. The transplanting was performed when the average air temperature was 15–20 °C, and the soil temperature was at least 15 °C. The climate in the growing location is typically Mediterranean. The plants were grown following conventional practices for the crop. Before transplantation, the soil was fertilized with phosphorous, and no additional fertilization was performed during cultivation. Watering was performed by drip irrigation. Weeds were mechanically removed by hoeing twice during the crop (no herbicide was used). No plant pruning was applied. Okra pods were harvested in July, when they reached fruit maturity. The same plant developmental stage and fruit maturity were considered for harvesting all the accessions. The harvest was performed early in the morning, and the pods were immediately taken to the Postharvest Physiology Laboratory of the Department of Horticulture, Faculty of Agriculture, Cukurova University, for phenotypic and biochemical analyses. Approximately 120 commercially acceptable (i.e., mature pods without visible signs of disease or physiological disorders) and uniform fruits per accession were selected based on size, color, and absence of defects. Fruits were divided into four replicates, each consisting of 30 fruits, for further quality analyses.

### 3.2. Fruit Morphometric Measurements

FFW and FDW were assessed by weighting fresh fruits and fruits dried at 60 °C until constant weight, respectively, using 30 fruits per replicate. DM was estimated based on the latter variables according to the following formula: DM = (FDW/FFW) × 100 and expressed as percentage of fresh weight (fw). Fruit diameters were measured from 10 fruits per replicate using digital calipers and expressed in mm. Each fruit was measured in two places, one adjacent to the stem and another at the center of the pod, and averaged to estimate fruit diameter. FL was measured with the same apparatus and expressed in mm.

### 3.3. Color Measurements

Color parameters were estimated with a tri-stimulus colorimeter (Minolta CR400, Konica Minolta Inc., Tokyo, Japan) in the CIE L*a*b mode. Color changes were quantified in the hue angle (h = 180 + tan − 1(b*/a*)), L* (luminance), and C* (chroma index) [30]. Thirty fruits from each of the four replicates were used for color measurements, and two measurements from the equatorial sides of each fruit were performed and averaged.

### 3.4. Chlorophyll Content

Approximately 0.1 g of peel was surgically removed with a razor blade to separate the exocarp from the mesocarp from 10 fruits per replicate. The peel was washed with distilled water for about 1 min and dried between sheets of filter paper. Chlorophyll was extracted from the fruit peels by the cold acetone 80% method described earlier [31]. Briefly, 0.1 g of sample was dissolved in 5 mL of acetone, homogenized, and then centrifuged at 5000 rpm for 5 min. The clear supernatant was transferred to a clean 5 mL tube, and spectrophotometric (Shimadzu Scientific Instruments, Columbia, MD, USA) absorbance measurements were taken at 663 nm (chlorophyll a) and 645 nm (chlorophyll b), according to Porra [31]. Total chlorophyll content was estimated as the sum of chlorophyll a and chlorophyll b. A solution of 80% acetone was used as a blank (control). Chlorophyll content was expressed as mg/100 g fw. This whole procedure takes ~15–20 min.

### 3.5. Ascorbic Acid Determinations

Ascorbic acid (i.e., vitamin C) was determined from 10 fruits per replicate following a protocol described earlier [32]. The fruits were weighed and put into a commercial blender. An equal weight of 0.1 N oxalic acid was then added to the blender and homogenized for 5 min. After blending, the solution was centrifuged, and the supernatant was recovered and filtered through a 1 µm glass fiber nylon filter. Prior to determinations, 1 mL of the okra extract was mixed with 1 mL of 2,6-dichlorophenol indophenol (DCIPP) and homogenized. Ascorbic acid in the sample was determined spectrophotometrically (Shimadzu Scientific Instruments, Columbia, MD) using a DCIPP solution. A series of ascorbic acid solutions in the range of 1–10 µg/mL were used as standards to generate calibration curves. Absorbance readings at 520 nm were taken, and the results were expressed as mg/100 g dw.

### 3.6. Statistical Analysis

Data were subjected to analysis of variance (ANOVA) carried out using SAS [33]. All values were standardized, and comparisons among the accessions were assessed using the Gower coefficient for mixed data [34] with the PAST package program (URL: https://past.en.lo4d.com/windows). A dendrogram and pair-wise dissimilarity heat map were constructed based on UPGMA [35].

## 4. Conclusions

The present work represents the most comprehensive evaluation of an okra germplasm collection based on fruit quality traits due to the large number (and diversity) of accessions and traits analyzed. Significant and substantial variation was found for all the traits, and superior genotypes were identified for morphometric and yield-related variables (FL, FD, FFW, FDW, and DM), chlorophyll and vitamin C contents, and color parameters. These materials are of value for okra breeding programs aiming at increasing fruit quality, postharvest conservation (as influenced by DM content), and nutraceutical value (e.g., by increasing chlorophyll and vitamin C levels), either for fresh consumption or processing. For example, accessions TR-42-1 and TR-77-1, exhibiting the greatest chlorophyll and vitamin C levels, could be selected for fresh consumption, while TR-35-1, which had the greatest DM content, may be used for long-term postharvest conservation and processing. In addition, the broad variation found for the majority of these traits will be instrumental in future research aiming at discovering the genetic factors conditioning relevant fruit attributes. Correlation analysis evidenced significant associations among 13 fruit quality traits, varying broadly in the strength and sign of the association. Phenotypic-based clustering analysis revealed clades with accessions that shared similar geographical origins and fruit traits. The distribution of Turkish materials across different clusters suggests broad phenotypic variation for these germplasm. These results complement previous characterizations of Turkish okra germplasm based on molecular markers, and there is good agreement for some clades between the genetic- and phenotypic-based analyses. It is expected that the data gathered in the present work will be useful for advancing breeding, germplasm curation, and genetic research in okra.

## Figures and Tables

**Table 1:** Mean values and standard errors for fruit fresh weight, dry weight, dry matter content, diameter, length, and peduncle diameter in the okra germplasm collection.

Accession	FFW (g)	FDW (g)	DM (%)	FD (mm)	FL (mm)	PD (mm)
TR–47–1	4.81 ± 0.31 e–q	0.62 ± 0.02 d–j	13.4 ± 0.7 bc	10.6 ± 0.4 o–v	45.8 ± 1.6 g–s	*4.0 ± 0.1 y*
TR–05–1	5.04 ± 0.42 d–q	0.70 ± 0.07 b–i	13.7 ± 0.6 bc	14.3 ± 0.3 c–e	37.7 ± 1.8 p–x	4.1 ± 0.1 w–y
TR–27–1	4.69 ± 0.19 g–q	0.54 ± 0.03 h–j	11.7 ± 0.8 c	12.4 ± 0.4 g–n	48.0 ± 2.7 e–p	4.7 ± 0.2 s–y
TR–17–1	5.32 ± 0.60 d–q	0.70 ± 0.08 b–i	13.4 ± 0.5 bc	11.0 ± 0.2 l–v	46.9 ± 2.3 g–q	4.7 ± 0.2 r–y
TR–17–2	4.66 ± 0.29 h–q	0.56 ± 0.04 g–j	12.2 ± 0.7 bc	10.4 ± 0.3 q–v	41.4 ± 2.6 k–v	5.0 ± 0.2 k–u
TR–35–1	4.39 ± 0.80 j–q	0.80 ± 0.15 b–g	**19.6 ± 1.6 a**	11.4 ± 0.3 j–u	55.2 ± 3.3 a–h	4.9 ± 0.3 m–u
TR–09–1	4.85 ± 0.35 e–q	0.65 ± 0.06 c–j	13.9 ± 1.5 bc	10.8 ± 0.4 m–v	40.7 ± 1.7 m–v	4.0 ± 0.1 x–y
TR–48–1	4.76 ± 0.46 f–q	0.65 ± 0.06 c–j	14.1 ± 0.7 bc	10.1 ± 0.3 r–v	48.5 ± 2.9 d–o	5.3 ± 0.2 g–s
TR–70–1	4.53 ± 0.37 i–q	0.53 ± 0.05 h–j	11.9 ± 0.8 c	12.1 ± 0.4 i–q	38.8 ± 1.3 o–w	4.5 ± 0.2 s–y
TR–42–1	5.74 ± 0.42 c–l	0.69 ± 0.08 b–i	11.8 ± 0.6 c	11.5 ± 0.2 j–u	29.7 ± 1.2 w–x	4.9 ± 0.2 o–v
TR–68–1	5.57 ± 0.29 c–m	0.67 ± 0.05 c–j	11.9 ± 0.5 c	12.4 ± 0.3 h–o	36.1 ± 2.0 r–x	6.1 ± 0.4 c–g
TR–64–1	4.61 ± 0.47 h–q	0.58 ± 0.07 e–j	12.9 ± 0.9 bc	10.7 ± 0.4 n–v	46.9 ± 4.0 g–q	5.7 ± 0.1 d–n
TR–60–1	4.83 ± 0.31 e–q	0.64 ± 0.06 c–j	11.8 ± 0.6 c	12.6 ± 0.8 f–m	*28.5 ± 2.2 x*	4.3 ± 0.1 u–y
TR–37–1	5.02 ± 0.16 d–q	0.59 ± 0.04 e–j	11.7 ± 0.1 c	11.7 ± 0.4 j–s	40.7 ± 2.9 l–v	5.3 ± 0.2 e–s
TR–54–1	4.53 ± 0.47 i–q	0.53 ± 0.06 h–j	11.0 ± 0.8 c	12.0 ± 0.6 i–q	**63.1 ± 5.8 a**	5.36 ± 0.3 g–s
TR–01–2	6.34 ± 0.46 c–g	0.71 ± 0.08 b–i	11.6 ± 0.8 c	11.4 ± 0.2 j–u	41.4 ± 2.7 k–v	5.3 ± 0.2 e–s
TR–01–3	5.32 ± 0.33 d–q	0.60 ± 0.04 e–j	11.2 ± 0.9 c	12.8 ± 1.4 e–k	39.2 ± 3.3 n–w	5.8 ± 0.3 c–l
TR–33–1	3.88 ± 0.25 p–q	*0.43 ± 0.04 j*	13.4 ± 1.0 bc	11.2 ± 0.3 k–u	43.0 ± 2.7 j–u	5.0 ± 0.2 l–u
TR–33–2	5.24 ± 0.17 d–q	0.70 ± 0.05 b–i	10.7 ± 0.7 c	11.6 ± 0.4 j–s	41.4 ± 2.7 k–v	5.3 ± 0.1 f–s
TR–01–4	6.29 ± 0.30 c–h	0.65 ± 0.04 c–j	11.3 ± 0.8 c	13.6 ± 0.4 d–i	55.9 ± 1.7 a–g	6.3 ± 0.3 b–d
TR–63–1	5.18 ± 0.43 d–q	0.61 ± 0.08 d–j	13.0 ± 0.7 bc	13.9 ± 0.5 d–h	42.7 ± 2.3 j–u	5.8 ± 0.2 c–k
TR–63–2	4.13 ± 0.41 l–q	0.51 ± 0.03 h–j	12.4 ± 0.5 bc	11.4 ± 0.4 j–u	39.9 ± 3.0 m–v	4.9 ± 0.1 o–w
TR–63–3	4.70 ±0.27 g–q	0.57 ± 0.02 g–j	13.7 ± 1.3 bc	12.2 ± 0.3 i–p	48.1 ± 1.4 e–p	4.8 ± 0.2 q–y
TR–Co–1	5.51 ± 0.54 c–o	0.72 ± 0.07 b–h	11.2 ± 0.5 c	12.9 ± 0.6 e–k	36.1 ± 3.5 r–x	4.8 ± 0.3 p–w
TR–10–1	5.72 ± 0.38 c–l	0.65 ± 0.06 c–j	11.6 ± 0.5 c	12.6 ± 0.6 f–m	34.6 ± 1.9 t–x	5.6 ± 0.2 d–p
TR–20–1	4.93 ± 0.32 e–q	0.58 ± 0.05 f–j	13.0 ± 0.8 bc	10.4 ± 0.3 q–v	43.8 ± 5.8 j–u	5.8 ± 0.2 c–k
Akkoy	3.89 ± 0.24 n–q	0.51 ± 0.05 h–j	17.0 ± 1.6 ab	*9.3 ± 0.4 v*	42.5 ± 3.2 j–v	4.9 ± 0.2 o–w
TR–48–2	5.01 ± 0.32 d–q	0.62 ± 0.05 d–j	13.2 ± 1.7 bc	10.4 ± 1.1 q–v	52.0 ± 4.3 b–j	5.6 ± 0.1 d–q
TR–05–2	5.44 ± 0.60 c–o	0.67 ± 0.09 c–j	11.5 ± 0.9 c	12.2 ± 0.4 i–p	45.4 ± 4.2 g–s	5.2 ± 0.2 h–s
TR–10–2	5.68 ± 0.42 c–l	0.67 ± 0.02 b–j	13.5 ± 1.1 bc	14.9 ± 0.5 b–d	46.6 ± 2.3 g–q	5.2 ± 0.2 h–s
TR–77–1	3.97 ± 0.24 m–q	0.53 ± 0.04 h–j	12.6 ± 1.4 bc	9.7 ± 0.4 u–v	51.6 ± 3.6 b–k	5.1 ± 0.3 i–u
TR–20–2	5.42 ± 0.56 c–p	0.64 ± 0.07 c–j	11.5 ± 0.6 c	13.7 ± 0.7 d–i	34.8 ± 2.8 t–x	5.1 ± 0.2 h–t
TR–20–3	5.57 ± 0.50 c–m	0.69 ± 0.07 b–i	12.2 ± 0.7 bc	14.1 ± 0.4 d–g	48.6 ± 3.5 d–o	5.6 ± 0.2 d–q
TR–15–1	5.11 ± 0.57 d–q	0.58 ± 0.06 f–j	12.3 ± 0.7 bc	15.7 ± 1.0 bc	47.6 ± 4.0 f–p	5.8 ± 0.1 c–k
TR–Co–2	4.53 ± 0.26 i–q	0.52 ± 0.03 h–j	12.3 ± 0.6 bc	9.9 ± 0.2 s–v	41.5 ± 1.8 k–v	5.0 ± 0.2 l–u
TR–Co–3	5.00 ± 0.52 d–q	0.71 ± 0.06 b–h	13.4 ± 1.1 bc	10.5 ± 0.2 p–v	42.7 ± 2.6 j–v	5.1 ± 0.2 h–t
TR–Co–4	4.83 ± 0.58 e–q	0.53 ± 0.08 h–j	13.6 ± 0.8 bc	10.8 ± 0.6 n–v	46.2 ± 3.2 g–r	4.7 ± 0.2 r–y
Cyprus	5.58 ± 0.48 c–m	0.70 ± 0.06 b–i	11.9 ± 0.5 c	11.2 ± 0.7 k–u	34.4 ± 2.6 u–x	**7.3 ± 0.3 a**
TR–35–2	4.80 ± 0.28 e–q	0.63 ± 0.02 d–j	12.1 ± 1.0 bc	10.6 ± 0.4 p–v	55.6 ± 4.7 a–h	5.3 ± 0.2 f–s
JA–1	4.90 ± 0.41 e–q	0.59 ± 0.05 e–j	12.4 ± 0.5 bc	10.9 ± 0.7 m–v	32.5 ± 1.4 v–x	5.2 ± 0.2 h–s
JA–2	4.29 ± 0.44 k–q	0.52 ± 0.03 h–j	13.5 ± 0.8 bc	11.0 ± 0.4 l–v	52.1 ± 3.0 b–j	5.9 ± 0.2 c–i
A–1	5.85 ± 0.24 c–k	0.70 ± 0.05 b–i	13.3 ± 0.5 bc	11.3 ± 0.3 j–u	45.8 ± 2.3 g–s	6.5 ± 0.3 bc
A–2	4.36 ± 0.23 j–q	0.57 ± 0.02 g–j	13.2 ± 0.5 bc	9.8 ± 0.3 t–v	49.3 ± 2.9 c–n	5.8 ± 0.3 c–k
A–3	5.39 ± 0.31 c–p	0.72 ± 0.05 b–h	12.3 ± 0.9 bc	10.2 ± 0.1 r–v	46.6 ± 2.3 g–q	6.1 ± 0.2 c–e
A–4	5.87 ± 0.44 c–k	0.67 ± 0.06 c–j	11.8 ± 0.5 c	12.8 ± 0.4 e–l	45.4 ± 1.9 h–s	5.9 ± 0.5 c–h
A–5	5.21 ± 0.63 d–q	0.58 ± 0.08 f–j	12.8 ± 0.7 bc	11.7 ± 0.3 j–s	57.8 ± 3.1 a–e	4.6 ± 0.2 s–y
A–6	5.46 ± 0.56 c–o	0.82 ± 0.06 b–f	11.0 ± 0.9 c	11.0 ± 0.4 l–v	52.1 ± 3.0 b–j	5.9 ± 0.2 c–i
A–7	5.60 ± 0.39 c–m	0.62 ± 0.06 d–j	13.9 ± 0.8 bc	9.8 ± 0.5 t–v	47.0 ± 4.3 g–q	4.4 ± 0.2 t–y
A–8	6.10 ± 0.37 c–i	0.81 ± 0.06 b–g	*10.5 ± 1.0 c*	9.8 ± 0.3 t–v	58.1 ± 3.6 a–d	5.1 ± 0.2 h–t
A–9	5.91 ± 0.22 c–k	0.75 ± 0.03 b–h	12.8 ± 0.7 bc	10.2 ± 0.3 r–v	46.4 ± 2.4 g–r	5.8 ± 0.3 c–k
A–10	5.39 ± 0.50 c–p	0.59 ± 0.05 e–j	11.53 ± 0.8 c	11.4 ± 0.3 j–u	41.1 ± 2.8 l–v	6.1 ± 0.3 c–f
A–11	5.70 ± 0.40 c–l	0.67 ± 0.05 c–j	15.5 ± 0.6 abc	11.5 ± 0.4 j–t	45.5 ± 2.5 h–s	5.6 ± 0.2 d–o
AF–1	6.99 ± 0.75 c	0.83 ± 0.11 b–e	12.4 ± 0.4 bc	10.9 ± 0.6 m–v	35.7 ± 3.4 s–x	5.8 ± 0.4 c–j
AF–2	8.29 ± 0.71 b	0.91 ± 0.06 b	12.6 ± 0.9 bc	16.2 ± 1.0 b	45.7 ± 3.6 g–s	5.7 ± 0.3 d–m
AF–3	**9.99 ± 0.96 a**	1.22 ± 0.17 a	12.9 ± 0.4 bc	**19.0 ± 0.9 a**	36.7 ± 2.0 q–x	6.8 ± 0.3 ab
AF–4	6.46 ± 0.46 c–e	0.85 ± 0.08 b–c	12.3 ± 0.4 bc	14.2 ± 0.3 d–f	44.7 ± 1.4 i–t	5.7 ± 0.3 d–n
AF–5	8.82 ± 1.30 ab	**1.34 ± 0.15 a**	12.8 ± 0.6 bc	14.4 ± 1.0 c–e	29.6 ± 2.0 w–x	5.5 ± 0.3 d–r
IN–1	5.41 ± 0.28 c–p	0.67 ± 0.03 c–j	12.5 ± 0.6 bc	11.9 ± 0.3 j–r	58.7 ± 2.8 a–c	5.1 ± 0.2 j–u
IN–2	5.66 ± 0.23 c–l	0.72 ± 0.04 b–h	11.5 ± 0.6 c	13.0 ± 0.4 e–j	54.3 ± 1.9 a–i	5.1 ± 0.2 j–u
IN–3	6.37 ± 0.21 c–f	0.71 ± 0.05 b–i	10.6 ± 0.9 c	11.5 ± 0.2 j–t	51.9 ± 2.9 b–j	5.1 ± 0.2 j–u
IN–4	6.63 ± 0.34 cd	0.80 ± 0.03 b–g	13.1 ± 0.6 bc	11.7 ± 0.5 j–s	39.1 ± 3.1 n–w	4.7 ± 0.2 s–y
IN–5	5.04 ± 0.29 d–q	0.69 ± 0.04 b–i	12.1 ± 0.8 bc	10.6 ± 0.1 o–v	50.1 ± 2.6 b–m	5.1 ± 0.2 j–u
IN–6	5.55 ± 0.37 c–n	0.68 ± 0.03 b–i	14.2 ± 0.8 bc	10.6 ± 0.3 p–v	47.0 ± 2.8 g–q	4.8 ± 0.2 p–x
IN–7	*3.76 ± 0.24 q*	0.46 ± 0.03 i–j	13.7 ± 0.5 bc	9.8 ± 0.2 t–v	43.0 ± 2.4 j–u	4.1 ± 0.1 v–y
IN–8	5.22 ± 0.29 d–q	0.68 ± 0.05 b–i	10.7 ± 0.8 c	11.8 ± 0.4 j–r	60.0 ± 2.2 ab	4.5 ± 0.2 s–y
IN–9	5.07 ± 0.35 d–q	0.62 ± 0.05 d–j	11.1 ± 0.8 c	10.8 ± 0.5 m–v	50.0 ± 3.0 b–m	5.2 ± 0.2 h–t
Mean ± SE	5.37 ± 0.41	0.67 ± 0.06	12.6 ± 0.8	11.8 ± 0.4	45.0 ± 2.8	5.3 ± 0.2
CV (%)	19.7	21.8	11.6	14.9	16.9	12.3

FFW: fruit fresh weight; FDW: fruit dry weight; DM: dry matter; FD: fruit diameter; FL: fruit length; PD: peduncle diameter. For each variable, the lowest and greatest mean values are indicated with italics and bold letters, respectively. Mean values within each column sharing the same letter are not statistically different at *p* < 0.05, Duncan test. Coefficient of variation (CV) values within each column are indicated in the last row.

**Table 2:** Mean values and standard errors for concentration of chlorophyll a (CAC), chlorophyll b (CBC), total chlorophyll (TCC), and vitamin C (Vit C), and the color parameters luminance (L*), hue angle (Hue), and chroma index (Chr) in the okra germplasm.

Accession	CAC	CBC	TCC	Vit C	L*	Hue	Chr
TR–47–1	1.17 ± 0.10 o–u	0.62 ± 0.15 j–p	1.86 ± 0.28 p–u	48.4 ± 2.3 h–q	51.7 ± 1.1 d–r	158.6 ± 27.8 b–d	574.0 ± 84.0 b–l
TR–05–1	2.34 ± 1.09 i–r	3.16 ± 0.61 b	5.50 ± 0.50 b–h	46.2 ± 4.0 i–q	40.3 ± 0.2 v–w	95.2 ± 4.7 c–d	684.8 ± 32.0 a–g
TR–27–1	0.89 ± 0.05s–u	0.52 ± 0.17 k–p	1.42 ± 0.23 s–u	*12.8 ± 2.4 z*	56.6 ± 1.6a–h	93.9 ± 7.3 c–d	362.5 ± 125.7 k–n
TR–17–1	3.89 ± 0.34 b–g	1.64 ± 0.16 d–j	5.52 ± 0.51 b–h	47.2 ± 3.0 i–q	56.9 ± 1.2 a–e	101.4 ± 3.9 c–d	395.6 ± 132.6 i–n
TR–17–2	3.84 ± 0.15 b–h	1.84 ± 0.17 d–i	5.68 ± 0.20 b–g	64.7 ± 1.8 b–e	51.9 ± 1.1 d–q	94.5 ± 5.4 c–d	723.8 ± 95.7 a–e
TR–35–1	2.51 ± 0.20 g–o	0.95 ± 0.08 g–p	3.47 ± 0.29 h–s	50.1 ± 2.1 g–q	55.2 ± 3.5 a–j	87.0 ± 9.5 d	511.9 ± 102.4 d–m
TR–09–1	4.70 ± 0.25 a–c	2.18 ± 0.32 b–e	6.88 ± 0.58 b–c	44.2 ± 3.7 i–s	42.1 ± 0.9 u–w	124.4 ± 25.1 b–d	653.2 ± 54.3 a–j
TR–48–1	2.29 ± 0.11 i–s	1.02 ± 0.04 g–p	3.30 ± 0.15 i–t	15.7 ± 1.7 y–z	51.3 ± 1.3 e–r	93.0 ± 6.5 c–d	604.3 ± 51.9 a–l
TR–70–1	0.96 ± 0.13 r–u	0.42 ± 0.08 m–p	1.38 ± 0.21 s–u	52.6 ± 0.8 e–n	49.4 ± 0.4 j–s	102.7 ± 4.1 c–d	**862.1 ± 26.9 a**
TR–42–1	**5.25 ± 0.17 a**	**4.06 ± 0.84 a**	**8.91 ± 1.21 a**	60.7 ± 1.5 c–h	54.8 ± 0.2 a–l	96.4 ± 0.4 c–d	570.0 ± 26.5 b–l
TR–68–1	2.88 ± 0.24 e–n	1.12 ± 0.03 e–p	4.01 ± 0.28 g–p	42.4 ± 1.7 j–t	49.3 ± 0.6 k–s	95.5 ± 5.4 c–d	760.6 ± 39.5 a–d
TR–64–1	3.09 ± 0.22 d–k	0.71 ± 0.38 j–p	3.80 ± 0.17 g–p	48.3 ± 1.9 h–q	58.8 ± 2.3 a	89.2 ± 15.5 c–d	254.7 ± 148.2 m–n
TR–37–1	1.78 ± 0.35 k–u	0.95 ± 0.23 g–p	2.72 ± 0.57 k–u	53.4 ± 1.6 d–l	55.9 ± 0.2 a–i	101.7 ± 3.8 c–d	433.4 ± 68.1 g–n
TR–54–1	1.85 ± 0.16 k–u	0.68 ± 0.10 j–p	2.54 ± 0.27 l–u	43.2 ± 15.2 j–s	56.6 ± 0.8 a–g	98.9 ± 6.4 c–d	535.4 ± 102.4 c–l
TR–01–2	2.89 ± 0.60 e–n	1.07 ± 0.26 g–p	3.96 ± 0.87 g–p	25.7 ± 2.9 v–y	50.7 ± 0.5 i–r	105.4 ± 0.1 c–d	758.6 ± 99.2 a–d
TR–01–3	1.89 ± 0.26 k–u	0.99 ± 0.14 g–p	2.89 ± 0.40 j–u	77.6 ± 2.6 a	57.2 ± 1.4 a–d	105.7 ± 0.2 c–d	381.1 ± 142.7 j–n
TR–33–1	3.42 ± 0.47 c–j	2.95 ± 0.22 bc	6.37 ± 0.26 b–e	72.2 ± 2.8 a–c	50.2 ± 0.5 i–r	101.2 ± 3.9 c–d	859.0 ± 40.4 a
TR–33–2	2.35 ± 0.2 i–q	1.61 ± 0.2 d–k	3.96 ± 0.4 g–p	44.8 ± 11.8 i–r	58.5 ± 1.4 ab	93.8 ± 7.1 c–d	345.5 ± 32.8 l–n
TR–01–4	2.06 ± 0.3 j–u	0.91 ± 0.2 g–p	2.96 ± 0.5 i–u	39.8 ± 2.2 n–u	52.0 ± 1.3 d–q	102.7 ± 3.2 c–d	708.5 ± 54.1 a–g
TR–63–1	2.55 ± 0.3 g–o	1.43 ± 0.4 d–n	3.98 ± 0.6 g–p	63.8 ± 1.9 b–e	54.8 ± 1.1 a–l	100.4 ± 5.3 c–d	455.0 ± 48.0 e–n
TR–63–2	1.79 ± 0.3 k–u	0.63 ± 0.1 j–p	2.42 ± 0.3 n–u	38.6 ± 3.7 p–u	49.0 ± 1.6 l–s	97.3 ± 7.8 c–d	694.6 ± 75.9 a–g
TR–63–3	1.77 ± 0.3 k–u	0.55 ± 0.1 j–p	2.32 ± 0.4 o–u	31.7 ± 2.2 s–x	49.7 ± 0.4 j–s	99.7 ± 5.6 c–d	673.9 ± 38.5 a–h
TR–Co–1	1.79 ± 0.1 k–u	0.73 ± 0.1 j–p	2.52 ± 0.2 l–u	13.5 ± 4.4 z	**59.7 ± 2.3 a**	105.7 ± 0.3 c–d	246.7 ± 147.1 n
TR–10–1	1.80 ± 0.3 k–u	0.81 ± 0.1 h–p	2.61 ± 0.4 l–u	60.8 ± 1.1 c–h	56.9 ± 0.9 a–e	96.1 ± 4.7 c–d	547.9 ± 137.4 b–l
TR–20–1	0.80 ± 0.1 t–u	0.34 ± 0.1n–p	1.14 ± 0.1 t–u	24.6 ± 9.7 w–z	56.7 ± 0.9 a–f	95.7 ± 4.7 c–d	567.3 ± 9.3 b–l
Akkoy	2.97 ± 0.3 d–l	0.86 ± 0.3 g–p	3.83 ± 0.6 g–p	42.4 ± 10.2 j–t	50.4 ± 1.5 i–r	98.7 ± 3.5 c–d	669.8 ± 34.0 a–h
TR–48–2	1.58 ± 0.2 l–u	0.61 ± 0.1 j–p	2.19 ± 0.2 o–u	52.4 ± 2.6 e–o	55.1 ± 0.9 a–k	96.4 ± 7.4 c–d	440.2 ± 63.4 f–n
TR–05–2	2.57 ± 0.3 g–o	1.18 ± 0.3 e–p	3.75 ± 0.6 g–r	53.2 ± 2.3 d–m	49.0 ± 1.0 l–s	84.4 ± 13.8 d	620.8 ± 62.7 a–k
TR–10–2	*0.76 ± 0.0 u*	0.34 ± 0.0 n–p	1.10 ± 0.1 u	53.8 ± 1.2 d–l	59.3 ± 0.9 a	89.7 ± 15.6 c–d	411.0 ± 62.9 h–n
TR–77–1	2.70 ± 0.7 f–n	1.88 ± 0.9 d–h	4.57 ± 1.6 e–n	**82.8 ± 3.2 a**	48.3 ± 1.1 n–t	96.9 ± 4.7 c–d	725.0 ± 15.0 a–e
TR–20–2	1.19 ± 0.5 o–u	0.48 ± 0.2 l–p	1.67 ± 0.6 q–u	64.2 ± 4.5 b–e	50.8 ± 0.7 h–r	95.3 ± 10.6 c–d	803.1 ± 97.2 a–c
TR–20–3	1.06 ± 0.1 p–u	0.49 ± 0.1 l–p	1.56 ± 0.3 r–u	28.5 ± 4.1 u–x	52.6 ± 0.1 c–o	95.6 ± 10.6 c–d	712.3 ± 136.8 a–f
TR–15–1	2.11 ± 0.9 j–u	1.11 ± 0.7 f–p	3.21 ± 1.7 i–u	55.4 ± 4.1 d–j	58.9 ± 2.8 a	*81.9 ± 13.7 d*	*239.5 ± 129.3 n*
TR–Co–2	3.99 ± 0.9 a–f	2.19 ± 0.7 b–e	6.17 ± 1.6 b–f	61.8 ± 3.6 c–g	52.9 ± 1.9 b–o	104.5 ± 0.4 c–d	600.7 ± 123.0 a–l
TR–Co–3	2.21 ± 0.9 i–t	0.47 ± 0.3 l–p	2.57 ± 1.3 l–u	33.0 ± 1.5 r–w	51.8 ± 0.4 d–q	104.5 ± 1.0 c–d	610.4 ± 38.0 a–l
TR–Co–4	1.51 ± 0.4 m–u	0.42 ± 0.0 m–p	1.93 ± 0.4 o–u	20.8 ± 5.2 x–z	59.0 ± 0.6 a	105.1 ± 0.3 c–d	383.9 ± 59.3 i–n
Cyprus	0.82 ± 0.1 t–u	0.26 ± 0.0 o–p	*1.07 ± 0.0 u*	50.4 ± 6.4 f–p	50.4 ± 1.6 i–r	96.0 ± 4.9 c–d	762.9 ± 123.8 a–d
TR–35–2	1.46 ± 0.0 n–u	0.57 ± 0.0 j–p	2.04 ± 0.0 o–u	56.5 ± 4.5 d–i	54.2 ± 1.2 a–n	105.8 ± 0.3 c–d	589.5 ± 107.3 a–l
JA–1	2.93 ± 0.3 d–m	1.15 ± 0.2 e–p	4.08 ± 0.5 f–o	48.4 ± 1.9 h–q	48.5 ± 1.5 m–t	105.3 ± 0.1 c–d	826.1 ± 69.2 ab
JA–2	1.93 ± 0.3 k–u	0.64 ± 0.1 j–p	2.57 ± 0.4 l–u	25.6 ± 3.6 v–z	54.3 ± 2.8 a–m	98.0 ± 3.7 c–d	826.6±37.4 ab
A–1	2.32 ± 0.1 i–r	0.92 ± 0.0 g–p	3.24 ± 0.1 i–u	42.0 ± 2.7 k–t	50.8 ± 3.7 g–r	104.7 ± 0.6 c–d	655.4 ± 25.3 a–i
A–2	3.06 ± 0.3 d–k	1.64 ± 0.3 d–j	4.70 ± 0.6 d–l	48.8 ± 0.2 h–q	55.2 ± 1.5 a–j	138.3 ± 39.2 b–d	576.0 ± 71.2 b–l
A–3	2.82 ± 0.1 e–n	0.38 ± 0.0 n–p	3.20 ± 0.1 i–u	55.3 ± 2.0 d–j	50.6 ± 0.8 i–r	101.7 ± 4.0 c–d	708.7 ± 41.9 a–g
A–4	4.30 ± 0.6 a–d	2.34 ± 0.2 b–d	6.64 ± 0.7 b–e	48.0 ± 0.3 h–q	46.6 ± 1.8 p–u	90.4 ± 7.6 c–d	642.9 ± 87.3 a–j
A–5	2.85 ± 0.2 e–n	1.02 ± 0.1 g–p	3.87 ± 0.3 g–p	45.5 ± 0.3 i–r	*19.0 ± 0.7 x*	452.1 ± 11.6 a	400.9 ± 22.6 h–n
A–6	1.16 ± 0.1 o–u	0.68 ± 0.2 j–p	1.84 ± 0.3 p–u	37.1 ± 4.4 q–v	48.4 ± 1.0 n–t	92.2 ± 7.08 c–d	685.7 ± 103.9 a–g
A–7	1.88 ± 0.1 k–u	0.81 ± 0.1 h–p	2.57 ± 0.2 l–u	64.8 ± 0.5 b–e	37.8 ± 6.5 w	**519.1 ± 147.7 a**	640.2 ± 62.9 a–j
A–8	3.13 ± 0.4 d–k	1.52 ± 0.2 d–m	4.65 ± 0.6 d–m	15.0 ± 0.4 y–z	52.2 ± 1.0 d–p	93.4 ± 7.6 c–d	645.9 ± 124.4 a–j
A–9	2.62 ± 0.6 f–n	0.94 ± 0.3 g–p	3.56 ± 0.9 g–s	55.3 ± 2.8 d–j	47.8 ± 1.4 o–t	101.6 ± 4.0 c–d	761.9 ± 20.1 a–d
A–10	0.92 ± 0.2 r–u	0.58 ± 0.2 j–p	1.5 ± 0.3 s–u	39.5 ± 2.1 o–u	46.4 ± 3.7 p–u	122.9 ± 57.6 b–d	731.7 ± 52.8 a–e
A–11	3.60 ± 0.6 b–i	1.55 ± 0.3 d–l	5.15 ± 0.8 c–i	53.3 ± 1.1 d–m	50.9 ± 0.2 f–r	94.7 ± 3.6 c–d	739.4 ± 25.5 a–d
AF–1	3.37 ± 0.4 c–j	0.76 ± 0.5 i–p	4.13 ± 0.5 f–o	40.3 ± 4.8 m–u	40.5 ± 1.3 v–w	103.8 ± 0.8 c–d	720.1 ± 24.6 a–e
AF–2	2.67 ± 0.4 f–n	1.08 ± 0.2 g–p	3.75 ± 0.6 g–r	66.0 ± 0.1 b–d	50.7 ± 0.7 i–r	94.0 ± 6.9 c–d	821.0 ± 35.7 ab
AF–3	1.88 ± 0.1 k–u	1.37 ± 0.7 d–n	2.53 ± 0.1 l–u	53.4 ± 1.6 d–l	50.9 ± 1.1 f–r	94.4 ± 5.2 c–d	802.6 ± 61.2 a–c
AF–4	3.44 ± 0.2 c–j	1.39 ± 0.1 d–n	4.83 ± 0.3 c–k	54.9 ± 2.5 d–k	40.3 ± 0.9 v–w	97.5 ± 7.7 c–d	637.5 ± 23.0 a–j
AF–5	1.92 ± 0.1 k–u	0.69 ± 0.1 j–p	2.61 ± 0.1 l–u	21.6 ± 0.9 w–z	46.3 ± 2.8 p–u	103.6 ± 0.4 c–d	823.6 ± 51.0 ab
IN–1	4.56 ± 0.6 a–c	2.36 ± 0.5 b–d	6.92 ± 1.0 b–c	63.0 ± 2.3 b–f	48.4 ± 0.2 n–t	91.9 ± 7.2 c–d	785.3 ± 54.2 a–d
IN–2	2.74 ± 0.5 f–n	1.07 ± 0.2 g–p	3.81 ± 0.7 g–p	30.5 ± 2.0 t–x	52.6 ± 0.6 c–o	95.7 ± 5.6 c–d	603.6 ± 58.8 a–l
IN–3	2.98 ± 0.2 d–l	1.01 ± 0.1 g–p	3.99 ± 0.3 g–p	42.8 ± 0.9 j–t	46.2 ± 0.2 q–u	90.7 ± 4.1 c–d	746.1 ± 18.4 a–d
IN–4	4.83 ± 0.1 ab	1.91 ± 0.1 e–g	6.73 ± 0.2 b–d	78.0 ± 1.6 a	48.6 ± 0.4 m–t	95.3 ± 5.4 c–d	827.3 ± 47.9 ab
IN–5	2.44 ± 0.6 h–p	0.99 ± 0.3 g–p	3.44 ± 0.8 h–s	50.3 ± 1.3 f–p	48.2 ± 0.5 o–t	96.0 ± 2.2 c–d	685.8 ± 73.8 a–g
IN–6	2.91 ± 0.4 e–m	0.43 ± 0.2 m–p	3.34 ± 0.6 h–s	46.5 ± 1.9 i–q	44.4 ± 2.8 s–v	200.8 ± 104.9 b	702.5 ± 67.9 a–g
IN–7	4.21 ± 0.7 a–e	2.18 ± 0.7 b–f	6.39 ± 1.4 b–e	19.8 ± 2.0 x–z	42.9 ± 0.9 t–w	101.1 ± 4.2 c–d	694.5 ± 25.6 a–g
IN–8	2.14 ± 0.4 j–u	*0.23 ± 0.0 p*	2.24 ± 0.5 o–u	41.0 ± 2.2 l–t	50.7 ± 1.3 i–r	90.4 ± 9.3 c–d	732.1 ± 73.3 a–e
IN–9	3.63 ± 0.1 b–i	1.43 ± 0.0 d–n	5.04 ± 0.1 c–j	75.0 ± 7.3 ab	45.8 ± 0.4 r–u	121.3 ± 25.3 b–d	770.3 ± 31.6 a–d
Mean ± SE	2.50 ± 0.32	1.14 ± 0.23	3.62 ± 0.49	46.8 ± 3.0	50.5 ± 1.3	113.6 ± 11.4	629.7 ± 67.1
CV (%)	42.8	65.6	46.8	34.8	12.7	60.9	25.2

CAC, CBC, and TCC are expressed as mg/100 g fw. Vit C content is expressed as mg/100 g dw. Values within each column sharing the same letter are not statistically different at *p* < 0.05, Duncan test. For each variable, the lowest and greatest mean values are indicated with italics and bold letters, respectively. Mean values within each column sharing the same letter are not statistically different at *p* < 0.05, Duncan test. Coefficient of variation (CV) values within each column are indicated in the last row.

**Table 3:** Correlation coefficient (r) values among 13 phenotypic traits in the okra germplasm collection.

Trait	FDW	FD	DM	FL	PD	CAC	CBC	TCC	Vit C	L*	Hue	Chr
**FFW**	0.89 ***	0.63 ***	−0.08	−0.23 *	0.42 **	0.02	−0.04	−0.04	0.01	−0.13	−0.01	0.25 **
**FDW**		0.51 ***	0.38 **	−0.20	0.32 *	0.01	−0.05	−0.04	−0.09	−0.11	−0.1	0.23
**FD**			−0.14	−0.25 *	0.27 *	−0.22	−0.01	−0.17	0.08	0.09	−0.17	−0.03
**DM**				0.07	−0.13	0.01	−0.02	0.01	−0.16	0.04	−0.19	−0.02
**FL**					−0.05	−0.04	−0.19	−0.10	−0.09	−0.04	0.15	−0.14
**PD**						−0.23	−0.24	−0.26 *	0.06	0.23	−0.27 *	0.11
**CAC**							0.76 ***	0.96 ***	0.29*	−0.26 **	−0.03	0.17
**CBC**								0.90 ***	0.33 **	−0.16	−0.08	0.11
**TCC**									0.32 **	−0.24	−0.05	0.15
**Vit C**										−0.08	0.1	0.16
L*											−0.62 ***	−0.40 **
**Hue**												−0.09

Fruit fresh weight, FFW; fruit dry weight, FDW; fruit diameter, FD; dry matter, DM; fruit length, FL; peduncle diameter, PD; chlorophyll a content, CAC; chlorophyll b content, CBC; total chlorophyll content, TCC; vitamin C, Vit C; chromas, Chr. Asterisks denote significant correlations at *p* < 0.05 (*), *p* < 0.01 (**), and *p* < 0.001 (***). Moderate-to-strong correlation values (*r* > 0.30) are indicated in bold letters.

**Table 4:** Code, name, seed source, and geographical origin of 66 okra accessions used in this study.

			Geographical Origin		
Code ^a^	Name	Seed Source	City	Province	Country
A-1	Cajun Queen	Di.Va.P.R.A ^e^	n.a.	n.a.	USA
A-2	Dixie Spineless	Di.Va.P.R.A	n.a.	n.a.	USA
A-3	Dwarf Long Good Green	Di.Va.P.R.A	n.a.	n.a.	USA
A-4	Lee	Di.Va.P.R.A	n.a.	n.a.	USA
A-5	Red Wonder	Di.Va.P.R.A	n.a.	n.a.	USA
A-6	Okra Brazil	Di.Va.P.R.A	n.a.	n.a.	USA
A-7	UGA Red Okra	Georgia Univ.	n.a.	n.a.	USA
A-8	Emerald	San Martin Seed Co.	n.a.	n.a.	USA
A-9	Perkins Spineless	Asgrow	n.a.	n.a.	USA
A-10	Jefferson	Univ. Arkansas	n.a.	n.a.	USA
A-11	Jade	Univ. Arkansas	n.a.	n.a.	USA
AF-1	803 Burkina Faso	IBPGR ^f^	n.a.	n.a.	Burkina Faso
AF-2	Red Balady	Ain Shams Univ.	n.a.	n.a.	Egypt
AF-3	1051 Togo	IBPGR	n.a.	n.a.	Togo
AF-4	2163 Sudan	IBPGR	n.a.	n.a.	Sudan
AF-5	1159 Togo	IBPGR	n.a.	n.a.	Togo
Akkoy	n.a.	n.a.	n.a.	n.a.	Turkey
Cyprus	n.a.	n.a.	n.a.	n.a.	Cyprus
IN-1	Vaishali Badhu	Di.Va.P.R.A	n.a.	n.a.	India
IN-2	Pusa Makhamali	Di.Va.P.R.A	n.a.	n.a.	India
IN-3	Vaishali Badhu	Di.Va.P.R.A	n.a.	n.a.	India
IN-4	PSM	Di.Va.P.R.A	n.a.	n.a.	India
IN-5	Tn.a.13	Di.Va.P.R.A	n.a.	n.a.	India
IN-6	Pakistana	Di.Va.P.R.A.	n.a.	n.a.	India
IN-7	Parbhani Kranti	ICRISAT ^g^	n.a.	n.a.	India
IN-8	Selectionn.a.2	NBPGR ^h^	n.a.	n.a.	India
IN-9	Arka Anamika	Maharashtra Seed	n.a.	n.a.	India
JA-1	Holiday	Gunma Prefecture	n.a.	n.a.	Japan
JA-2	Japan	Hyogo Prefecture	n.a.	n.a.	Japan
TR-01-2	n.a.	n.a.	Karsıyaka	Adana	Turkey
TR-01-3	n.a.	n.a.	Aladag	Adana	Turkey
TR-01-4	n.a.	n.a.	Balcalı	Adana	Turkey
TR-05-1	TR48520	AARI ^b^	Amasya	Amasya	Turkey
TR-05-2	n.a.	ACHRI ^c^	Yalova	Yalova	Turkey
TR-09-1	TR57359	AARI	Aydın	Aydın	Turkey
TR-10-1	n.a.	n.a.	Balıkesir	Balıkesir	Turkey
TR-10-2	n.a.	ACHRI	Yalova	Yalova	Turkey
TR-15-1	n.a.	PDA ^d^	Burdur	Burdur	Turkey
TR-17-1	TR43092	AARI	Canakkale	Canakkale	Turkey
TR-17-2	TR43099	AARI	Canakkale	Canakkale	Turkey
TR-20-1	n.a.	n.a.	Denizli	Denizli	Turkey
TR-20-2	n.a.	ACHRI	Yalova	Yalova	Turkey
TR-20-3	n.a.	ACHRI	Yalova	Yalova	Turkey
TR-27-1	TR40293	AARI	Gaziantep	Gaziantep	Turkey
TR-33-1	n.a.	n.a.	Alata	Mersin	Turkey
TR-33-2	n.a.	n.a.	Tarsus	Mersin	Turkey
TR-35-1	TR57346	AARI	Izmir	Izmir	Turkey
TR-35-2	n.a.	n.a.	İzmir	İzmir	Turkey
TR-37-1	TR51605	AARI	Kastamonu	Kastamonu	Turkey
TR-42-1	TR69231	AARI	Konya	Konya	Turkey
TR-47-1	TR35291	AARI	Mardin	Mardin	Turkey
TR-48-1	TR61730	AARI	Muğla	Muğla	Turkey
TR-48-2	n.a.	n.a.	Dalaman	Mugla	Turkey
TR-54-1	TR68542	AARI	Sakarya	Sakarya	Turkey
TR-60-1	TR51691	AARI	Tokat	Tokat	Turkey
TR-63-1	n.a.	n.a.	Sanlıurfa	Sanlıurfa	Turkey
TR-63-2	n.a.	n.a.	Sanlıurfa	Sanlıurfa	Turkey
TR-63-3	n.a.	n.a.	Sanlıurfa	Sanlıurfa	Turkey
TR-64-1	TR66581	AARI	Usak	Usak	Turkey
TR-68-1	TR69234	AARI	Aksaray	Aksaray	Turkey
TR-70-1	TR69229	AARI	Karaman	Karaman	Turkey
TR-77-1	n.a.	ACHRI	Yalova	Yalova	Turkey
TR-Co-1	n.a.	n.a.	Marmara	Marmara	Turkey
TR-Co-2	n.a.	n.a.	May Tohumculuk	May Tohumculuk	Turkey
TR-Co-3	n.a.	n.a.	Sultani Mey	Sultani Mey	Turkey
TR-Co-4	n.a.	n.a.	Karaburun	Karaburun	Turkey

^a^ The code for each accession is as follows: country or continent abbreviation (A: America; AF: Africa; IN: India; TR: Turkey), plate number of the province (only for Turkish accessions), and collection number. ^b^ Aegean Agricultural Research Institute, ^c^ Ataturk Central Horticultural Research Institute, ^d^ Provincial Agricultural Directorate, ^e^ Department of Exploitation and of Agricultural and Forestry Resources, ^f^ International Board for Plant Genetic Resources, ^g^ International Crops Research Institute for the Semi-Arid Tropics, ^h^ National Bureau of Plant Genetic Resources.

## Data Availability

The original contributions presented in this study are included in the article. Further inquiries can be directed to the corresponding authors.
